# RETRACTION: Effects of Bundle‐Care Interventions on Pressure Ulcers in Patients with Stroke: A Meta‐Analysis

**DOI:** 10.1111/iwj.70288

**Published:** 2025-03-03

## Abstract

**RETRACTION:** WangL.‐P. , GaoM.‐M. , WangX.‐Q. , GuM.‐M. , and QiQ.‐D. , “Effects of Bundle‐Care Interventions on Pressure Ulcers in Patients with Stroke: A Meta‐Analysis,” International Wound Journal 21, no. 2 (2024): e14432, 10.1111/iwj.14432.PMC1082871337853846

The above article, published online on 18 October 2023, in Wiley Online Library (http://onlinelibrary.wiley.com/), has been retracted by agreement between the journal Editor in Chief, Professor Keith Harding; and John Wiley & Sons Ltd. Following an investigation by the publisher, all parties have concluded that this article was accepted solely on the basis of a compromised peer review process. The editors have therefore decided to retract the article. The authors did not respond to our notice regarding the retraction.

**RETRACTION:** L.‐P. Wang , M.‐M. Gao , X.‐Q. Wang , M.‐M. Gu , and Q.‐D. Qi , “Effects of Bundle‐Care Interventions on Pressure Ulcers in Patients with Stroke: A Meta‐Analysis,” International Wound Journal 21, no. 2 (2024): e14432, 10.1111/iwj.14432.PMC1082871337853846

The above article, published online on 18 October 2023, in Wiley Online Library (http://onlinelibrary.wiley.com/), has been retracted by agreement between the journal Editor in Chief, Professor Keith Harding; and John Wiley & Sons Ltd. Following an investigation by the publisher, all parties have concluded that this article was accepted solely on the basis of a compromised peer review process. The editors have therefore decided to retract the article. The authors did not respond to our notice regarding the retraction.

